# Inhibition of IGF signalling pathway in MDA-MB-231 triple negative breast cancer cells

**DOI:** 10.1186/1753-6561-6-S4-O14

**Published:** 2012-07-09

**Authors:** S Chandran, JH Harmey, S Toomey

**Affiliations:** 1 School of Medicine, Royal College of Surgeons in Ireland; 2 Molecular and Cellular Therapeutics, Royal College of Surgeons in Ireland

## Introduction

Triple-negative breast cancer (TNBC) is characterised by the absence of estrogen receptor (ER), progesterone receptor (PR) and the HER-2 receptor. TNBC is typically associated with a poor prognosis due to aggressive tumour phenotypes, partial response to chemotherapy, and current lack of clinically validated targeted therapies. Insulin-like growth factors (IGFs) stimulate cell proliferation and promote cell survival via receptor phosphorylation and activation of adaptor proteins such as mitogen-activated protein kinase (MAPK), and Akt. The overall aim of this project was to characterise the expression and activation of the IGF signalling pathway in the MDA-MB-231 TNBC cell line.

## Methods

Expression of steroid and growth hormones and activation of the IGF signalling pathway in MDA-MB-231 cells was analysed by western blotting, while expression of PAPP-A was detected by PCR. The effect of stimulation with IGF1 or inhibition of EGFR/IGF1R tyrosine kinase activity on proliferation was determined by the MTS cell proliferation assay. Proliferation was expressed relative to untreated controls, and data was analysed by ANOVA with Tukey's multiple comparison post hoc test.

## Results

MDA-MB-231 cells express epidermal growth factor receptor (EGFR) and low levels of HER3, and were confirmed as negative for ER, PR and HER2. IGFBP4 inhibits IGF1 activity but cleavage by pregnancy associated plasma protein A (PAPP-A) protease releases active IGF1. MDA-MB-231 cells express high levels of insulin-like growth factor binding protein 4 (IGFBP4), and low levels of PAPP-A. Moreover, MDA-MB-231 cells express type I IGF1 receptor and proteins along the IGF signalling cascade; namely, Erk and Akt. The presence of phosphorylated forms of these proteins shows activation of the IGF1R signal transduction pathway in MDA-MB-231 cells. Proliferation was increased by IGF1 (E3R) (recombinant IGF1, resistant to binding by IGFBPs). Inhibition of EGFR tyrosine kinase activity or IGF1R tyrosine kinase activity inhibited proliferation of MDA-MB-231 cells and a similar effect was observed with dual inhibitors of PI3K/mTOR or Akt/ P70S6K.

## Conclusions

These results suggest that the IGF1 signalling pathway is activated in MDA-MB-231 TNBC cells. Therefore, inhibition of the IGF1R and/or its downstream targets may be of benefit in the treatment of TNBC.

